# Concept recognition for extracting protein interaction relations from biomedical text

**DOI:** 10.1186/gb-2008-9-s2-s9

**Published:** 2008-09-01

**Authors:** William A Baumgartner, Zhiyong Lu, Helen L Johnson, J Gregory Caporaso, Jesse Paquette, Anna Lindemann, Elizabeth K White, Olga Medvedeva, K Bretonnel Cohen, Lawrence Hunter

**Affiliations:** 1 Center for Computational Pharmacology, University of Colorado School of Medicine, 12801 E. 17th Ave Aurora, Colorado, 80045, USA

## Abstract

**Background:**

Reliable information extraction applications have been a long sought goal of the biomedical text mining community, a goal that if reached would provide valuable tools to benchside biologists in their increasingly difficult task of assimilating the knowledge contained in the biomedical literature. We present an integrated approach to concept recognition in biomedical text. Concept recognition provides key information that has been largely missing from previous biomedical information extraction efforts, namely direct links to well defined knowledge resources that explicitly cement the concept's semantics. The BioCreative II tasks discussed in this special issue have provided a unique opportunity to demonstrate the effectiveness of concept recognition in the field of biomedical language processing.

**Results:**

Through the modular construction of a protein interaction relation extraction system, we present several use cases of concept recognition in biomedical text, and relate these use cases to potential uses by the benchside biologist.

**Conclusion:**

Current information extraction technologies are approaching performance standards at which concept recognition can begin to deliver high quality data to the benchside biologist. Our system is available as part of the BioCreative Meta-Server project and on the internet .

## Background

Early efforts in information extraction have focused primarily on identification of character strings and, for the most part, have not been adopted for use by biologists. We posit that a prominent factor in the biologist's reluctance to rely on current information extraction technologies is the ambiguity that remains in these extracted strings of text. For example, there are a multitude of tools that can extract gene names from text. This is a classic problem in biomedical natural language processing (BioNLP), and one that has been extensively studied [1,2]. Determining that a particular string of text in a larger document corresponds to a gene name is a challenging problem, and by no means one that should be discounted. However, from a biologist's perspective, knowing that a string of characters is a gene name leaves much to be desired. Among other things, it would be helpful to know exactly which gene and from which species the identified character string is referring. This phenomenon is not limited to the identification of gene names in text, but applies also to many of the common targets of biomedical information extraction, such as cell types, diseases, tissues, and so on.

Recently, however, efforts have shifted toward the identification of concepts as opposed to character strings [3]. Concepts differ from character strings in that they are grounded in well defined knowledge resources. Concept recognition provides the key piece of information missing from a string of text - an unambiguous semantic representation of what the characters denote. The BioCreative II tasks have provided a platform to evaluate concept recognition systems in the field of biomedical language processing. As a demonstration of the potential effectiveness of integrating concept recognition, we have constructed a protein interaction relation extraction system, the components of which were generated through participation in several of the BioCreative II tasks.

We took a modular approach to the BioCreative II tasks, building on system components from other tasks whenever possible. To facilitate component integration, we made extensive use of the Unstructured Information Management Architecture (UIMA) [4,5] framework. Four benefits accrued from this strategy. First, the complete integration of all processing steps allowed us to experiment quickly and easily with different approaches to the many subtasks involved. Second, it made it easy for us to evaluate quickly the results of these experiments against the official datasets. Third, it provided us with a clean interface for incorporating tools from other groups, including LingPipe [6], A Biomedical Named Entity Recognizer (ABNER) [7], and Schwartz and Hearst's abbreviation detection algorithm [8]. Finally, it allowed for distribution of workload over the construction of the various system components that were created.

A key focus in our work, and for the protein-protein interaction extraction task (interaction pair subtask [IPS]) in particular, was the use of a concept recognition system being developed by our group. Called Open source Direct Memory Access Parser (OpenDMAP), it is a modern implementation of the DMAP paradigm first developed by Riesbeck [9], Martin [10], and Fitzgerald [11]. The earliest descriptions of the paradigm assumed that a DMAP system would approach all levels of linguistic analysis through a single optimization procedure. In this work we show that analysis can be modularized, and even externalized, without losing the essential semantic flavor of the DMAP paradigm. Hunter and coworkers [3] have described OpenDMAP in detail.

It should be noted that the benefits of concept recognition are not limited to information extraction tasks. Concept recognition has the potential to also contribute to such areas of BioNLP as document retrieval and summarization. We will touch briefly on these during our discussion of the interaction article subtask (IAS) and the interaction sentence subtask (ISS), respectively.

## Identifying the concepts: the gene mention task

The first step to using concepts in any information extraction task is to determine the types of concepts that will be necessary to identify the named entities of interest. Our ultimate goal for this demonstration is to capture evidence of specific protein-protein interactions from text. The concepts we will primarily be interested in are genes and their products. This foundational step is analogous to the gene finding task in the biological domain. Once one has found the genes in a sequence, then one can focus on the more complex task of determining the function of the genes, how they are regulated, and so on. Similarly, once gene mentions have been identified in text, then further analyses can be performed, for example, to determine whether the genes and gene products are known to interact or participate in protein transport, or what cell types they are expressed in. The BioCreative II gene mention (GM) task provided a laboratory (of sorts) for developing the foundation on which to build more complex applications that rely on reliable GM detection. Identification of gene names (or mentions) in text is a classic problem in the field of BioNLP, and one that has been studied extensively [1]. Smith and coworkers [2] provided a detailed overview of the challenges of this task (also known as 'gene tagging') and the state-of-the-art in GM identification techniques. Although the central dogma of molecular biology clearly defines the difference between genes and proteins, it is important to note that ambiguity arises when genes and proteins are written about, so much so that biological experts often do not agree when a piece of text is referring to a gene or protein [12]. Throughout this paper, when speaking of extracting mentions from text, we use the words 'gene' and 'protein' interchangeably.

Our approach to GM identification focuses on the use of publicly available gene tagging systems and simple consensus approaches for combining their outputs. Baumgartner and coworkers [13] also described this methodology. We used two publicly available gene taggers [6,7] and a gene tagger developed in-house for the inaugural BioCreative GM task [14].

Two general strategies for combining gene tagger output were used to test two distinct hypotheses. Our first hypothesis, the 'consensus hypothesis', posited that filtering the output of multiple gene taggers by requiring agreement by two or more of the individual systems would result in an overall precision measure greater than or equal to the highest precision measure of the individual systems. Our second hypothesis, the 'combining hypothesis', posited that combining the output of multiple gene taggers would result in an overall recall measure greater than or equal to the highest recall measure of the individual systems.

We implemented two methods for combining the output of multiple gene taggers to test these hypotheses. The Consensus Hypothesis was tested by building a consensus-based filter with variable thresholds for consensus determination. This filter implements a simple voting scheme in which each tagger is given an equal vote. We then varied the consensus threshold from three (all taggers agree) to two (two of the three taggers agree). If a particular gene mention accumulates the required threshold of votes, then it is kept. If the threshold is not met, the gene mention is not returned. To test the Combining Hypothesis, we implemented a filter that keeps all gene mentions labeled by the individual taggers. Unlike the consensus filter, the combining filter attempts to deal with issues of differing boundaries in the outputs of the individual taggers. When two gene mentions are found to overlap, the filter keeps the longer gene mention and discards the other, a decision motivated by the results of BioCreative 2004 GM systems that extended gene mention boundaries [1].

### Gene mention results

When evaluated against the GM task held-out test data, the results were consistent with both hypotheses (Table 1). The consensus filter approaches were observed to elevate precision over any of the individual gene taggers. The combining filter also behaved as expected, by increasing the aggregate system's overall recall measure, with the consequence being a noticeable loss in precision. In comparison with the performances noted on the training data, the performances on the test data were considerably lower. We believe that this is an artifact of using the individual gene tagging systems as they came 'out-of-the-box'. All three gene taggers used models trained on data released during the BioCreative I GM task. The BioCreative I GM data are largely equivalent to the training data used during the BioCreative II GM task; Smith and coworkers [2] described changes made before BioCreative II to clean up the corpus and make the annotations more consistent. As a result of this similarity, symptoms of over-training were observed, in particular for the LingPipe system, which appeared to be trained on all of the available BioCreative I data. It is this over-training that we feel is largely responsible for the decrease in performance observed when evaluating against the test dataset.

**Table 1:** GM results: performances of systems and individual components on the test and training data.

	Test Data			Training Data		
Tagger	Precision	Recall	F-measure	Precision	Recall	F-measure

CCP	77.30	77.74	77.52	83.68	83.48	83.58
ABNER	80.38	73.26	76.65	83.85	80.86	82.33
LingPipe	72.53	80.00	76.09	88.47	92.77	90.57
2/3 Majority	85.54 (2)	76.83 (3)	80.95 (3)	91.15	86.33	88.68
Unanimous	92.78 (1)	49.12 (4)	64.24 (4)	94.56	61.41	74.46
Overlap	66.22 (4)	83.72 (2)	73.94 (4)	79.27	91.17	84.80
Median	85.08	79.05	81.32			

Presented are median scores, as supplied by organizers. Quartiles for our runs are shown in parentheses. GM, gene mention.

The question of the optimal number of gene tagging systems to use for this approach remains uninvestigated. However, our findings suggest that as few as three systems are sufficient for gearing a gene mention identification system toward either maximizing precision or maximizing recall, and therefore would enable a user to fine tune a system to the task at hand. Although the decision to abstain from retraining on the BioCreative II training data undoubtedly negatively affected our performance in this evaluation, we feel that our approaches still present an interesting argument for combining the output of multiple tools constructed for a similar task. Furthermore, we have demonstrated that reliable GM identification is within reach, paving the way for more complex information extraction tasks that rely on the identification of genes.

## Grounding the concepts: the gene normalization task

Our experiments for the GM task have demonstrated the ability to identify gene mentions in text at a relatively high level of accuracy. Identification of mentions in text, as we noted above, is only part of the concept recognition process. In order to truly recognize a concept, it is necessary to normalize (or ground) the mention to a unique entity in a well defined knowledge source. This knowledge source typically takes the form of a genomic database (for example, grounding a gene mention to a particular Entrez Gene [15] identifier) or a biological ontology (for example, associating a mention describing a molecular function with a particular Gene Ontology [16] concept). Attempts to address this problem have been met with limited success in the past [17,18] for a variety of reasons, species ambiguity being a prominent issue. The 2006 gene normalization (GN) task took steps to isolate the normalization problem by removing from the equation the often confounding question of species identification. By limiting the normalization procedure to human genes only, development efforts were able to focus solely on the task of mapping a gene mention to a lexicon of genes, namely the Entrez Gene database. It is important to note, however, that the ability to identify the species under discussion is just as important in the normalization procedure as mapping the mention to a particular gene. Elimination of this part of the GN task increases the feasibility of the task considerably. See the report by Morgan and coworkers [19] for further details on the BioCreative II GN task.

Our approach to the GN task builds upon work completed for the GM task. Briefly, gene mentions are identified and then processed into a regularized form. An attempt to find a unique Entrez Gene entry to map to is made, and if multiple entries are found then a disambiguation procedure is invoked. The primary novelty of our approach lies in the steps that we take to deal with resolving conjunctive structures. An iterative process was used during development of this system, whereby the system was evaluated on the entire development corpus (provided by the BioCreative organizers), then modified, and then evaluated again. This process was repeated until the developer was satisfied with the performance of the system. The results shown in the tables below reflect performance measures obtained during a *post hoc *analysis of the system. Analogous to knocking out a gene in a mouse and observing the phenotypic changes that occur, various components of the system were disabled to see how the overall performance was affected. The common performance measure shared by Tables 2 to 5 is therefore the best performance achieved by the system when evaluated against the development data.

**Table 2:** GN results: performance on the development data with and without conjunction resolution.

Steps	Precision	Recall	F measure
Without conjunction resolution	0.836	0.691	0.757
With conjunction resolution	0.827	0.727	0.774

GN, gene normalization.

**Table 3:** GN results: performance on the development data using different online resources for lexicon construction.

Resources	Genes entries	Precision	Recall	F measure
Entrez Gene	21,206	0.827	0.727	0.774
UniProt	18,580	0.834	0.591	0.692
Entrez Gene + UniProt	24,182	0.827	0.708	0.762

GN, gene normalization.

**Table 4:** GN results: performance impact of the seven heuristics used to normalize gene names on the development data.

	Rule	Example	*P*	R	F
0			0.783	0.469	0.586
1	Substitution: Roman letters > arabic numerals	carbonic andydrase XI to carbonic andydrase 11	0.778	0.492	0.603
2	Substitution: Greek letters > single letters	AP-2alpha to AP-2a	0.779	0.497	0.607
3	Normalization of case	CAMK2A to camk2a	0.787	0.619	0.693
4	Removal: parenthesized materials	sialyltransferase 1 (beta-galactoside alpha-2,6-sialytransferase) to sialyltransferase 1	0.782	0.623	0.694
5	Removal: punctuation	VLA-2 to VLA2	0.768	0.667	0.714
6	Removal: spaces	calcineurin B to calcineurinB	0.784	0.742	0.762
7	Removal: strings < 2 characters	P	0.827	0.727	0.774

Presented are the seven heuristics used to normalize gene names in both lexicon construction and during processing of the gene tagger output, and the performance on the development data after each step was performed. GN, gene normalization.

**Table 5:** GN results: performance with and without the gene name disambiguation procedure when evaluated against the development data.

Steps	Precision	Recall	F measure
Without disambiguation	0.848	0.689	0.760
Use abbreviations only	0.825	0.722	0.770
Use abbreviations and flanking regions	0.827	0.727	0.774

GN, gene normalization.

### Gene mention detection

Our GM identification component was tuned toward maximizing recall by using the combining filter developed for the GM task. We used six separate GM systems [6,7,14,20,21] (both the BioCreative 2004 and the Natural Language Processing in Biomedicine and its Applications[NLPBA] models were used with the ABNER system [7]) in conjunction with the combining filter. After manually examining the output from the development data, we developed a set of nine heuristics (Table 6) that were designed to either filter out obvious false positives, modify gene mentions so that they could be more easily matched to a dictionary of gene names, or both. Application of all nine heuristics resulted in the removal or modification of 1,086 putative gene mentions, and an increase in precision from 0.770 to 0.829 and in recall from 0.673 to 0.725 on the GN task development data. Two of the heuristics (rules 4 and 5) resulted only in the modification of the gene names, while one rule (rule 9) resulted in the removal of a gene name if a nonhuman keyword is detected, or modification of the gene name if it begins with a lowercase h (for example hCB2 [PubMedID: 8679694]). All other rules resulted in the removal of potential false-positive gene mentions.

**Table 6:** GN results: performance of nine heuristics used to filter false-positive gene mentions or modify gene mentions to improve dictionary matching performance.

	Presence of ...	Example	*P*	R	F	Modified
0			0.770	0.673	0.718	0
1	Gene chromosome location	3p11-3p12.1	0.772	0.673	0.719	34
2	Single, short lowercase word	heme	0.778	0.672	0.721	112
3	Strings of only numbers &/or punct	9+/-76	0.779	0.672	0.722	206
4	Extra preceding words	protein SNF to SNF	0.790	0.681	0.731	225^a^
5	Extra trailing words	SNF protein to SNF	0.812	0.723	0.765	419^a^
6	Amino acids	Ser-119	0.815	0.723	0.766	460
7	Protein families	Bcl-2 family proteins	0.816	0.722	0.766	701
8	Protein domains, motifs, fusion	SNH domain	0.828	0.722	0.771	883
9	Nonhuman keywords	rat IFN gamma	0.829	0.725	0.774	1,086^a^

Results depicted here are from the development dataset. Step 0 indicates performance before application of any rules. At each step, the rules of preceding steps are also applied. Modified refers to the cumulative number of gene mentions removed or altered. ^a^Rules 4 and 5 result in modification of gene mentions only. Rule 9 can result in either modification or removal of gene mentions. All other rules result in removal of gene mentions. GN, gene normalization.

### Conjunction resolution

Resolution of complex coordination is an unsolved problem in general, and in BioNLP it is particularly difficult to overcome because of the multitude of unconventional representations observed in biomedical text. Recently, Buyko and coworkers [22] proposed a means for dealing with coordination ellipses within biological named entities. For the GN task at hand, we noted the need to address conjunctive structures; approximately 8% (52/640) of gene names in the development dataset contained conjunctions, either of the general English (for example, HMG1 and 2) or domain-specific (for example, IL3/5) variety. To address these issues we developed a straightforward procedure for extracting individual gene names from some of the common conjunction types observed in the development dataset: gene names in regular coordinated structures (for example, IL3/IL5 refers to IL3 and IL5); gene names in a series (for example, freac1-freac7 refers to freac1, freac2, freac3 ... freac7); gene subtypes trailing the gene name (for example, IL3/5 refers to IL3 and IL5); and gene subtypes appearing before the gene name (for example, M and B creatine kinase refers to M creatine kinase and B creatine kinase).

The algorithm first looks for two typical conjunction-indicating words ('and' and 'to') and two atypical, domain-specific conjunction-indicating forms ('/' [forward slash] and '-' [hyphen]). Then, the algorithm builds the individual gene names from the conjoined structure. (See Lu [23] for further details.)

Table 2 shows the overall improvement in performance on the training data yielded by the conjunction resolution step. It is slight (the F measure increased only from 0.757 to 0.774), even though about 8% of gene tokens in the data appear in structures requiring some processing. One reason for this minor gain in performance is that some of the conjoined genes are also mentioned individually within the same document, allowing for their normalization outside of the conjunctive structure. Another reason is that some conjunctions were beyond the scope of our algorithm (for example granulocyte [G-] and granulocyte-macrophage [GM-] colony-stimulating factor [CSF]). Demonstration of at least a minimal capacity for handling coordinated structures is an important step, however, because this issue is commonplace in the field of BioNLP, and must be addressed, as we discuss below.

### Dictionary construction

Our GN algorithm relies on a lexicon of gene names as a target for matching identified gene mentions. To create this lexicon, we extracted the gene symbol, synonyms, and full name from Entrez Gene (Homo_sapiens.ags.gz file available at [24]) for each human gene. In addition to Entrez Gene, we also investigated other databases such as UniProt [25] and a combination of the two databases. We found the Entrez Gene database to be the best resource for gene dictionary construction for the current task (Table 3). This result is consistent with the conclusions reported by Cohen [26].

Examination of the dictionary entries showed that some entries could be removed without affecting system performance because they are of no use for GN tasks. See the report by Baumgartner and coworkers [13] for details on the exclusion of these gene entries. The dictionary used for the GN task contained 21,206 entries.

### Gene mention regularization

A set of heuristics was used to regularize all gene names and symbols in the dictionary and all gene mentions outputted by the GM system. These heuristics are based on earlier work [26,27] and on previous dictionary-based systems [28]. Table 4 shows the effects of the individual rules on performance. Use of all seven rules in sequential order resulted in a noticeable increase in F measure from 0.586 to 0.774.

### Mapping mentions to Entrez Gene identifiers

After the extracted gene mentions have been regularized and conjunctions have been addressed, the processed mentions are compared with all entries in the dictionary using exact string matching. If multiple matches are found, then a disambiguation procedure (discussed below) is invoked.

In addition to exact string matching, we also investigated some approximate string matching techniques. Like Fang and coworkers [28], we found that approximate matching noticeably increased search time but did not markedly improve performance.

### Gene name disambiguation

Gene names and symbols can be ambiguous across species when identical names and/or symbols are used to refer to orthologous genes, or within a species when a gene name or symbol is used to represent more than one distinct gene. For example, CHED is used as a synonym for two separate Entrez Gene entries: CHED1 (EntrezGene: 8197) and CDC2L5 (EntrezGene: 8621). Because the species question has been essentially removed from the equation for the task described here, we are concerned with only the latter. It has been estimated that more than 5% of terms for a single organism are ambiguous and that approximately 85% of terms are ambiguous across species [29,30]. For the (single-species) GN task, we developed two approaches to gene name disambiguation. The first method attempts to identify 'definitions' of gene symbols, using the Schwartz and Hearst algorithm [8], which identifies abbreviations and their long forms in text. Our second approach, similar to that of Lesk [31], examines the five tokens that appear before and after the ambiguous gene. We then calculate the Dice coefficient between both the abbreviation definitions and flanking tokens and the full name of each gene candidate, as given in Entrez Gene. The gene with the highest nonzero Dice coefficient is returned. If the Dice coefficients are all zero, we return nothing.

Our results indicate that finding unabbreviated gene names or flanking words plays an important role in resolving ambiguous terms (Table 5). Moreover, this gene name disambiguation procedure can provide evidence for a term being a false gene mention. For example, STS (PMID: 11210186) is recognized as a gene mention, but its surrounding words, *content mapping*, and *RH (Radiation Hybrid) analysis *indicate that it is an experimental method. We assembled a list of words suggesting nonprotein terms such as *sequence* or *analysis*. When they were matched to a gene's unabbreviated name or its flanking words, the gene was considered a false mention.

Even with the improvement yielded by this disambiguation procedure, gene name ambiguity remains a key contributor to system error. On the development data, our precision for mentions that only matched a single Entrez entry was 0.85, whereas for ambiguous entries it was only 0.63. (Recall is difficult to compute for the two cases, because we do not know how many mentions in the gold standard are ambiguous.)

### Other techniques applied

To further enhance system performance, especially with regard to false-positive gene mention identification, we assembled stop word lists consisting of common English words, protein family terms, nonprotein molecules, and experimental methods. The common English stop word list included 5,000 words derived by word frequency in the Brown corpus [32]. The protein family terms were derived from an in-house manual annotation project, which annotated protein families. A list of small molecules (for example, Ca) was also added. Words found in these lists were never recognized as gene names, even if they appear in the gene dictionary.

### Gene normalization results

Performance for three different runs submitted for the GN task are shown in Table 7. The F measure for all three runs is comparable to the highest F measure (0.79) for the GN task in mouse (the most comparable of the three species examined) in the BioCreative I GN task [17].

**Table 7:** GN results: performance on the GN test data.

Run	True positives	False positives	False negatives	Precision	Recall	F measure	Quartile
1	576	109	209	0.841	0.734	0.784	1
2	583	120	202	0.829	0.743	0.784	1
3	587	129	198	0.820	0.748	0.782	1

GN, gene normalization.

The ability of our system to handle simple cases of complex coordination as well as the straightforward mention regularization procedure and disambiguation techniques provide the basis for the concept recognition system that we will use for the more complex task of identifying and extracting protein interaction relations.

## Concept recognition for document retrieval: the protein interaction article subtask

There are various levels of extracted information that a bench biologist may be interested in. On a scale from broad to narrow in terms of scope, types of information extracted may be documents, informative sentences, and specific relations. The default choice for searching for documents relevant to a particular topic is PubMed. An alternative to the search engine approach is a machine learning (ML) approach, in which a statistical model of a document class is constructed and then used to find similar documents. The disadvantage to using a ML approach is the need for training data with which to build the model. However, if such training data are available, then the ML approach has the advantage of potentially identifying features in the text that are relevant to a particular class of documents but perhaps not obvious to the reader. For the IAS task, we take this ML approach, employing mainly linguistic features extracted from the training data.

Although some manner of concept recognition is used for this task in the form of interactions detected by our IPS system (described below), concept recognition is not used to its fullest extent and, as we discuss at the end of this section, turns out to be a weakness in our methodology. We use this example to demonstrate how the absence of concept recognition detrimentally affects the performance of our system.

We utilized the WEKA toolkit [33] to construct the ML-based classifiers. The linguistic features employed were *n*-grams (with *n *ranging from 1 to 5) of stemmed words. The conceptual features used were the presence/absence of protein interaction relations extracted by the system described in the IPS section (see below). Table 8 summarizes the characteristics of the three classifiers that were built. An attempt to balance the number of positive and negative examples in the training data was made for one of the classifiers, but the effect of this balancing was inconclusive. The report by Baumgartner and coworkers [13] provides further details of this aspect of our system.

**Table 8:** IAS methods: the three classifiers used for the IAS subtask.

Name	Classifier		IG threshold
Run 1	SVM	RBF kernel, complexity factor 100, gamma 0.001	0.0001
Run 2	Naïve Bayes	kernel estimation enabled	0.001
Run 3	SVM with balanced ±	RBF kernel, complexity factor 100, gamma 0.001	0.0001

IG threshold is the information gain feature selection threshold. IAS, interaction article subtask; RBF, radial basis function; SVM, support vector machine.

### Interaction article subtask results

All three classifiers constructed achieved F measures roughly equivalent to one another, and above, but within one standard deviation of, the overall mean performances for all groups that participated. As in our cross-validation experiments on the training data, our first run achieved the best F measure on the test data, but the difference in F measures between the three are relatively small. The support vector machine classifiers (runs 1 and 3) appear to achieve a higher precision and lower recall than the Naïve Bayes classifier, a characteristic that we have noticed in other document classification work where we compared these classification algorithms [34].

Interestingly, however, we note that our IAS classifiers achieved much higher performance in cross-validation on training data than on the test data. For example, our run 1 classifier achieved a precision of 0.951, a recall of 0.945, and an F measure of 0.948 in tenfold cross-validation of training data, which is a difference of 0.20 when compared with the F measure achieved on the test data. Cross-validation experiments are designed to minimize the effects of over-fitting, and our past experiences suggest that it is typically more successful than was indicated by this experiment. This suggests that a difference exists between the data compiled for the training set and the test set.

We analyzed the corpora and found that the publication years of the articles in the different sets showed that all of the positive training articles were published in either 2005 or 2006, whereas the negative training articles came from a wider distribution of publication years, centered around 2001. Only about 10% of the negative training articles were published in 2005 or 2006, and so it is possible that our classifiers discriminated partially based on the publication years (possibly represented in the feature sets, for example, by a bias in the types of experimental procedures mentioned). Our run 1 system expressed a bias toward positive classification on the test set (458 positive classifications and 292 negative classifications), where 91% of the articles were published in 2006.

Cohen and coworkers [35] noted a similar phenomenon in the Text Retrieval Conference (TREC) 2004 Genomics track data. Our analysis supports the findings of their more extensive study. The difference that they noted was substantially smaller than the one that we report here - about 12% versus the approximately 20% that we report - suggesting that the training/test data for the BioCreative IAS task might represent a good dataset for working on this problem.

Although this apparent publication year bias appears to be an issue with the construction of the training and test corpora, it brings to light a real-world problem that warrants our attention. The most likely use for document classification systems is to train on currently available data, and apply the model to literature as it is published. However, this approach requires that the system not be affected by the type of bias observed in our IAS study. A concept-based approach, in which terms are recognized and mapped to an ontology, as opposed to a purely linguistic-based approach, as we employed for these classifiers for the most part, might help avoid over-fitting of classifiers to development datasets. For example, we found that terms describing experimental approaches for detecting protein-protein interactions (for example, yeast two hybrid, two-dimensional gel electrophoresis, co-immunoprecipitation, and matrix-assisted laser desorption ionization time-of-flight mass spectrometry[MALDI-TOF]) were among the most important features in discriminating positive from negative articles. The useful information in these features is not the mention of a specific experimental method, but the fact that a technique for recognizing protein-protein interactions was mentioned. (As one reviewer of our workshop paper [13] suggested, this points out the value of having a knowledge model that reflects the curation criteria for the reference databases, because they only contain experimentally confirmed interactions.) This is consistent with the hypothesis (advanced by us and others elsewhere, for example [26,34,36]) that a better approach when training classifiers is to attempt to map words to their underlying concepts. Using this approach, we hypothesize that future systems would be more scalable and robust.

## Concept recognition for sentence retrieval: the protein interaction sentences subtask

The next level of increasing scope a bench biologist may be interested in is extraction of information at the sentence level. Sentences with high information content could potentially be used as a document summarization methodology, thus saving researchers time needed to hunt through articles to find these nuggets of a particular type of information. We, in fact, modeled the ISS subtask as a summarization task, using an approach similar to the Edmundsonian paradigm [37]; we created a scoring scheme to rate sentences as either containing an interaction mention, or not. This approach has also been used for selecting candidate GeneRIFs (Gene References Into Function) from Medline abstracts [38], which are analogous to sentences with high information content.

Development of our ISS system was based on a development set of 29 full-text articles with 53 gold standard sentences selected by IntAct [39] and MINT [40] database curators [41]. The system was iteratively adjusted by using feedback from evaluating on this development dataset.

### Sentence selection and scoring

The selection of a candidate interaction sentence is a two step process. The sentence must first meet certain eligibility requirements before it can be scored and considered a potential interaction sentence. The criteria for gauging eligibility and the features used for scoring the sentence are different as shown in Table 9. The eligibility and scoring criteria also differ depending on the location of the sentence in the document (section-specific usefulness and error rates have been noted in other BioNLP application areas, for example [21]).

**Table 9:** ISS methods: scoring requirements.

	Requires					Scored on				
Location	P	N	G	X	I	P	N	G	X	I
Abstract		×	×						×	×
Figure/table caption		×	×	×	×				×	×
Section/subsection heading		×			×				×	×
Other^a^		×	×		×	×			×	×

Column definitions: P = has positive cue words; N = does not have negative cue words; G = has > 0 gene mentions; X = has experimental methods; I = has interaction key word. ^a^If a sentence includes a reference to a figure or table, the score for the caption is added to the score for the sentence. ISS, interaction sentence subtask.

When a sentence is deemed eligible for scoring, a straightforward calculation is made to determine the score. For each mention of an experimental method for detecting protein-protein interaction that is present in the sentence, ten points are added to the sentence score. For each interaction keyword or positive cue term observed in the sentence, the number of points added to the sentence score equals the number of tokens present in the keyword or term observed. Here, we define token as any string of characters separated by white space in the interaction keyword or positive cue term. If a sentence includes a reference to a figure or table, then the score for the referenced figure or table caption is added to the score for the sentence. The lists of cue words, interaction keywords, and experimental methods are available upon request.

### Scoreable features

Our system scores each sentence in a full-text article with respect to the following features:

1. Frequent words. Words used with high frequency in the gold standard sentences are likely to be related to protein-protein interaction. For instance, the word 'interact' and the phrase 'interaction of' are the most frequent unigram and bigram, respectively.

2. Location. Most gold standard passages are located in the results section, and a few in the title, abstract, or introduction sections. Some sections are never observed to yield a sentence.

3. Mentions of gene/protein names. Because the sentences make assertions about protein-protein interaction, protein mentions are a necessary component of these sentences.

4. Summary-indicative cue words. These are words (for example, 'confirm') or phrases (for example, 'data establish') that indicate that a sentence is likely to be an information-rich sentence.

5. Mentions of experimental methods. Protein-protein interaction detection methods (for example, two hybrid array) are frequently mentioned in the gold standard passages.

6. Figure/table mention. Many gold standard passages reference a table or figure.

### Preprocessing

The methods used for HTML parsing and gene name tagging were the same as used for the IPS task (see the following section). In an attempt to remove false positives before processing, we implemented a document zoning filter that excluded sentences associated with certain document sections. The excluded document sections were chosen from manual inspection of the training data. The sections include the following: materials and methods, acknowledgments, discussion, reference, table of contents, disclosures, and glossary.

### Interaction sentence subtask results

We submitted two runs for the ISS task. The runs differed only in the passage length returned for each 'interaction sentence'. For our first submission, the returned passage was limited to a single sentence. This restriction was loosened for the second submission, permitting multiple consecutive high-scoring sentences to be returned. Our results show that loosening the passage length restriction permitted the extraction of 39.2% more passages than had been preselected by the human curators when compared with our single-sentence run (Table 10). This suggests that informative sentences regarding protein interactions in full text are likely to be found in close proximity. This contrasts with the case of abstracts, in which such sentences tend to be found at opposite ends of the text [38].

**Table 10:** ISS results: interaction passages extracted from the ISS task test data.

Run	Passages	TP	Unique	U_TP	TP/Passages	U_TP/Unique	MRR
Run #1	372	51	361	51	13.71	14.13	1.0
Run #2	372	71	361	70	19.09	19.39	1.0

Column headings: Passages = the total number of passages evaluated; TP = the number of evaluated passages that were preselected by human curators; Unique = the number of unique passages evaluated; U_TP, the number of unique passages that were pre-selected; MRR = mean reciprocal rank of the correct passages. ISS, interaction sentence subtask.

## Concept recognition for relation extraction: the protein interaction pairs subtask

Finally, the most detailed level of information that may interest a bench biologist is the extracted interaction data itself. This information could be presented to the biologist as the results of a literature search. Alternatively, the methods used to extract the data could be used to support database expansion and management. For the IPS subtask [42] we used OpenDMAP, which is a concept recognition system that has been developed by our group. As is typical for concept recognizers using manually constructed grammars, our system is geared toward optimizing precision. The procedure begins with preprocessing the HTML, and then moves to species recognition, entity tagging and part of speech tagging, followed by extraction of protein-protein interactions. Our approach for detecting interacting protein pairs relies heavily on the systems generated for the GM and GN tasks.

### Preprocessing

#### HTML parsing

The HTML parser developed to process the raw HTML documents was an extension of a similar parser developed for the TREC Genomics 2006 task [36]. The title, abstract, paragraphs, sentences, section headings, and subsection headings were extracted for each document. Document sections were inferred based on the section heading text. Sentence boundaries were detected using the LingPipe sentence chunker [6]. Sentences were mapped back to the original HTML using a dynamic programming approach.

#### Protein mention tagging

We used a variant of the system developed for the GM task to tag genes/proteins in which the outputs of ABNER [7] (both models) and LingPipe [6] (BioCreative04 model) were combined using the combining filter (see the section on GM, above). As we pointed out in the GM task introduction, the distinction between gene and protein mentions in text is often vague, and therefore for the purposes of the analyses conducted in this paper we consider them to be equivalent.

#### Linguistic tagging

Part of speech (POS) tagging was done using the GENIA POS Tagger [43].

#### Species classification

Species classification was done using a modified dictionary search. The species dictionary was constructed from the intersection of words from the National Center for Biotechnology Information (NCBI) names.dmp file (a list of all known scientific names and synonyms for organisms) and the set of NCBI taxonomy identifiers present in the IPS training set. These words were then combined into a single regular expression pattern for each species. In the flanking region of ± 50 characters around each detected species, we searched for bigrams that would further indicate a particular species in order to filter out false positive identifications. This set of 'indicator bigrams' was created by calculating the frequency of bigrams in the flanking region of the IPS training data. Each indicator bigram was assigned a log-odds score using the formula:

$$\frac{\text{P(bigram TP species match)}}{\text{P(bigram FP species match)}}$$

Log-odds scores were summed to determine the score of a single species match. The total score for a given species classification for a single article was calculated by combining the number of times a species match was made and the sum of the log-odds for indicator bigrams per match. Once scored, the species for a given document was returned in rank order. We experimented with the optimal number of species results to return and found the best results when the maximum number of species returned from the ranked list was two.

### Protein mention normalization

#### Gene/protein lexicon construction

Dictionaries were constructed for each species that was observed in the IPS training data by extracting information from the uniprot_light_table_updated.txt file supplied by the BioCreative organizers.

#### Protein mention normalization

Each gene/protein mention was normalized using the procedure described above for the GN task, using the dictionary for the identified species. We experimented with the optimal number of normalized identifiers to return and found the best results when we limited the output to one normalized entry per gene mention in text.

### OpenDMAP and conceptual patterns

We extracted protein-protein interaction pairs by applying OpenDMAP [3], an open source, ontology-based concept recognition system available at [44]. It works by associating manually written patterns to concepts in free text. The patterns combine information about concepts, keywords, parts of speech, phrase types, and other syntactic features into single patterns.

OpenDMAP patterns are written in a regular grammar syntax that consists of nonterminal elements on the left-hand side and terminal and nonterminal elements on the right. Nonterminal elements are linked to a Protégé ontology [45], which describes the protein-protein interaction frame with an interaction class that has two slots: interactor1 and interactor2. An example of an OpenDMAP pattern for the IPS task looks like the following expression:

{interaction} = [interactor1] interacts with [interactor2]

Where elements presented in {braces} represent classes in the ontology, elements in [brackets] correspond to slots of the class on the left-hand side of the pattern, and bare strings are terminals. The slots are constrained in the ontology to have specific features; for the IPS task, the slot elements [interactor1] and [interactor2] are constrained to be proteins.

**Figure 1:**
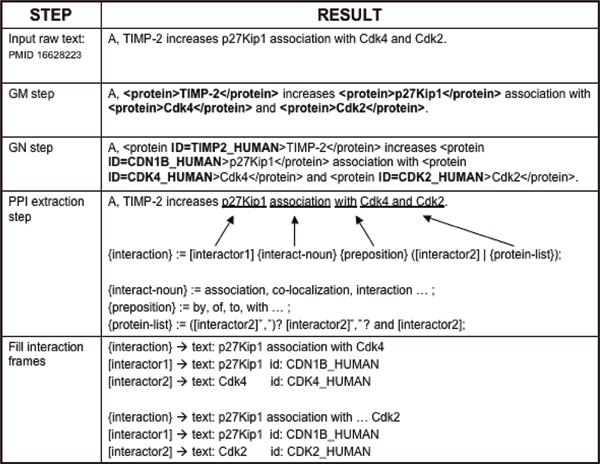
IPS: steps of the protein-protein interaction extraction system. IPS, interaction pair subtask.

When a sentence is input to the system, OpenDMAP recognizes that the marked proteins tagged by our GM system match the constraints on the frame slots [interactor1] and [interactor2]. When OpenDMAP matches the rest of the pattern elements, an instance of a protein-protein interaction frame is created. The interactor1 and interactor2 slots are filled with the protein instances from text that matched the pattern. The output is a protein-protein interaction frame from the ontology, filled in with instances of the interactors found in the text. See Figure 1 for a step-by-step representation of this process.

We used a variety of discovery procedures to build the patterns, including interview sessions with 'native speakers' (scientists with expertise in biology), and examination of corpora for pattern elements. The interviews were used to determine the set of predicates that described protein-protein interaction. Biologists were given a set of sentences in varying constructions (active, passive, and so on) and asked to determine whether plugging in the verbs from a list would result in a sentence denoting physical protein-protein interaction.

The corpus investigation uncovered frequently occurring *n*-grams and frequently occurring strings between protein mentions [46]. We used the BioCreative 2006 IPS, ISS, and IAS training data; the PICorpus (available at [47]) [48,49]; material generated by Jörg Hakenberg [50] and Anna Veuthey; and the Prodisen corpus (available at [51]).

The final grammar consisted of 67 rules. The patterns used in the IPS task are available at [44]. The grammar handles verbal and nominalization constructions, and various forms of conjunction, but not negation. We experimented with using unbounded wildcards, the results of which were higher recall but very low precision. We also experimented with the insertion of various parts of speech and phrase types between the protein slot pattern elements, with the result that the final pattern set includes adjective, adverb, and determiner POS elements, as well as various prepositional phrase types.

## Results

There was a marked difference between our performance on the training data and on the test data. Our results on the training data were *P *= 0.364, R = 0.044, and F = 0.078, returning 385 pairs. However, we achieved recall as high as 0.31 on the test data (seven times higher than on the training data), and recall higher than the median on two of five measures (see Tables 11 and 12). Our F measure was above the median more often than it was below it.

**Table 11:** IPS results: comparison of interaction pairs results on the IPS task test data.

	Calculated by interaction			Calculated by article		
	*P*	R	F	*P*	R	F

Run 1	0.38	0.06	0.11	0.39	0.31	0.29
Task median	0.06	0.11	0.07	0.08	0.20	0.08

'Calculated by interaction' indicates each interaction pair extracted was given equal weight. 'Calculated by article' indicates the measure was calculated by averaging over articles. Run1 was tuned to maximize precision. IPS, interaction pair subtask.

**Table 12:** IPS results: comparison of normalization results on the IPS task test data.

	Calculated by interactor			Calculated by article			Calculated by article with interactions		
	*P*	R	F	*P*	R	F	*P*	R	F

Run 1	0.57	0.12	0.19	0.15	0.13	0.13	0.56	0.46	0.48
Median	0.18	0.25	0.19	0.16	0.28	0.17	0.21	0.39	0.24

'Calculated by interactor' indicates each interactor extracted was given equal weight. 'Calculated by article' means the measure was calculated by averaging over articles. 'Calculated by article with interactions' means that only articles in which at least a single prediction submitted by a team was considered in the calculation. IPS, interaction pair subtask.

Analysis of the submission data show that 60% of the true positive protein-protein interaction assertions were expressed with a nominalization phrase, and 40% were expressed with a verbal phrase. By far the most common predicate was 'interact/interaction'; 66% of the nominalized PPI assertions and 39% of the verbal PPI assertions employed this predicate. The predicates used in the remainder of the assertions were spread among 11 other predicates, as shown in Table 13.

**Table 13:** IPS results: distribution of the predicates of the true positive protein-protein interactions extracted from the IPS task test data.

Verbs/nominalizations	Predicate	Count
Verbs	Interact	26
	Co-localize	9
	Bind	7
	Regulate	7
	Inhibit	6
	Associate	5
	Co-immunoprecipitate	2
	Suppress	2
	Co-precipitate	2
	Modulate	1
		
	Total	67
		
Nominalizations	Interaction	68
	Association	29
	Binding	20
	Co-localization	2
	Phosphorylation	1
		
	Total	103

IPS, interaction pair subtask.

Results for the normalization portion of this task are shown in Table 12. The precision of the method is competitive at 57%. The normalization recall of 12%, however, is detrimental for the output of the system as a whole. Note that the results of the gene normalization portion of the PPI task are far lower than those of the GN task because of the extra step of species disambiguation required in the former.

## Discussion

One goal of this work was to extend the OpenDMAP concept recognition system. We were able to do so, incorporating a number of third-party linguistic and semantic analysis tools without surrendering an essential characteristic of the DMAP paradigm: complete integration of semantic and linguistic knowledge, without segregating lexical and domain knowledge into separate components.

Our use of UIMA [4,5] as a framework for integrating the various software components used throughout our BioCreative II submissions was integral to the performances we were able to achieve. For each major component, a UIMA wrapper was created so that it could be plugged into the system. By using a standardized framework, we were not only able to distribute the tasks of development with the assurance that the pieces would work in concert once combined, but we were also able to design our systems in such a way that as they became successively more complicated, evaluation remained quick, easy, and modular. Not only was it possible to incorporate infrastructure constructed expressly for the BioCreative tasks, but it was just as easy to utilize external tools developed before the BioCreative tasks and/or by third-parties. This allowed us to benefit from LingPipe, Schwartz and Hearst's abbreviation-defining algorithm, ABNER, KeX, ABGene, and the GENIA POS tagger (op cit). Utilizing this framework provided not only a robust development architecture and production-ready execution environment, but also tremendous time savings.

The major goal of our work on this shared task, however, was to explore the integration of concept recognition in biomedical information extraction systems. The potential for information extraction is undeniable. As the breadth of knowledge in the biomedical literature continues to expand, it has become increasingly difficult for a single person to keep up with even a single specific research topic. Concept recognition techniques provide a potential remedy for this situation. As we discussed in the IAS section, the use of conceptual features could greatly benefit information retrieval as well as document classification systems. For the case of classifying protein interaction documents, defining a concept for 'experimental protein interaction detection methods' could potentially resolve some of the bias we encountered due to differences in the publication years among the training and test sets. It should be noted that there have been some contradictory reports on the benefits of using concepts, in particular in the domain of information retrieval. Results from the TREC Genomics *ad hoc *retrieval task in 2003 [52] pointed to the use of multiple concepts - MeSH headings, substance name fields in Medline, and species - as accounting for elevated performance. On the contrary, results from TREC Genomics 2004 [53] indicated that "[retrieval] approaches that attempted to map to controlled vocabulary terms did not fare as well." For proponents of concept recognition, this may first appear mildly disconcerting, but a closer examination of the TREC Genomics 2004 findings shows a number of factors that may be responsible for the poor performance. In particular, the systems classified as 'conceptual' simply were not very good at concept recognition, or made a poor choice of concepts by relying solely on a single conceptual type. In short, the role of concepts in these systems is somewhat overstated; thus, the conclusions regarding the influence of concept use should be tempered.

Integrating concept recognition into tasks other than information retrieval or document classification also has direct implications for the benchside biologist, among others. The merging of the many genomic databases by creating new links among their respective entities has the potential to uncover previously unknown information, or make known information more accessible to a wider population of scientists. The ultimate goal of extracting different relation types from text and generating links among concepts could be the potential for hypothesis generation and testing over the known 'facts' of biomedicine. This is certainly a lofty goal, but concept recognition is a key component to achieving automatic hypothesis generation and testing, and we, as a community, have taken the first steps down this path.

As detection of currently untapped conceptual types improves, so will the benefits of integrating conceptual recognition into current information gathering technologies. The BioCreative II tasks have provided a snapshot of the state of conceptual recognition in BioNLP, and all indications are that progress is being made. However, the potential of conceptually based systems will not be fully realized until concepts can be accurately, reliably, and unambiguously extracted from text.

## Abbreviations

ABNER, A Biomedical Named Entity Recognizer; BioNLP, biomedical natural language processing; GM, gene mention; GN, gene normalization; IAS, interaction article subtask; IPS, interaction pair subtask; ISS, interaction sentence subtask; ML, machine learning; NCBI, National Center for Biotechnology Information; OpenDMAP, Open source Direct Memory Access Parser; POS, part of speech; TREC, Text Retrieval Conference; UIMA, Unstructured Information Management Architecture.

## Competing interests

The authors declare that they have no competing interests.

## Authors' contributions

All authors contributed to the writing of the workshop proceedings paper. WAB, HLJ, and KBC were the main contributors to the writing of this paper. WAB developed and implemented the GM task methodologies, coded the UIMA infrastructure used in all tasks, and developed the HTML parsing code used in the ISS and IPS tasks. ZL developed and implemented the GN and ISS task methodologies. HLJ oversaw the execution of IPS subtask, including writing the OpenDMAP patterns, and analyzing the IPS results. JGC mentored AL and oversaw the execution of the IAS subtask and analyzed IAS results, and interviewed biologists for the IPS task. JP developed the species classifier used in the IPS subtask. AL constructed classifiers used in the IAS subtask. EKW extracted gene names from UniProt and compiled the GN dictionary files used for the ISS and IPS subtasks, as well as did corpus analysis for the IPS task. OM wrote the parser for the GN training data. KBC managed the team, coordinated and supervised the integration of the various tasks, and provided linguistic and software design contributions during all phases of the project. LH conceived of the project and supervised all aspects of it.
